# Supplementary material for: Ulnar finger posture effect on a pinch strength
Source: PLoS One. 2025 Jun 3;20(6):e0325359. doi: 10.1371/journal.pone.0325359 (PMC12133165; doi:10.1371/journal.pone.0325359)
Supplement: S1 Table — (DOCX) [file pone.0325359.s001.docx]

# Supporting Information

## S1 Table. Result of pinch strength in the natural ulnar finger posture

|  | | Natural ulnar finger posture | |
| --- | --- | --- | --- |
|  |  | Flexion | Extension |
| Ulnar finger posture | Hand dominance |  |  |
| Flexion | Dominant | 5.1 ± 1.9 | 3.9 ± 1.5 |
|  | Non-dominant | 4.7 ± 1.5 | 3.6 ± 1.1 |
| Extension | Dominant | 3.7 ± 1.6 | 3.2 ± 1.1 |
|  | Non-dominant | 3.2 ± 1.0 | 3.0 ± 1.1 |
| Data are presented as mean ± standard deviation (SD). Unit of the pinch strength is kilograms of force (kgf).  The posture during the initial pinching movement was defined as the “natural ulnar finger posture.” When the movement was performed in flexion initially, the natural ulnar finger posture was represented as “Flexion,” while when it was performed in extension initially, it was represented as “Extension.” | | | |
