# Supplementary material for: Ulnar finger posture effect on a pinch strength
Source: PLoS One. 2025 Jun 3;20(6):e0325359. doi: 10.1371/journal.pone.0325359 (PMC12133165; doi:10.1371/journal.pone.0325359)
Supplement: S2 Table — (DOCX) [file pone.0325359.s002.docx]

# Supporting Information

## S2 Table. Correlation of pinch strength ratio (flexion / extension) with participant’s demographic data.

| Participants’ demographic data | | Pinch strength ratio (flexion / extension) | |
| --- | --- | --- | --- |
|  |  | r | P-value |
| Age (years old) | | 0.05 | 0.779 |
| Height (cm) | | -0.15 | 0.398 |
| Body mass (kg) | | -0.07 | 0.681 |
| Hand size (cm) | Length | 0.24 | 0.186 |
|  | Width | -0.01 | 0.945 |
|  | Span | 0.08 | 0.656 |
|  | Middle finger length | 0.02 | 0.899 |
|  | Circumference | -0.03 | 0.890 |
| r = correlation coefficient  Note: the r value was calculated using a Peason’s correlation coefficient. | | | |
