# Supplementary material for: Ulnar finger posture effect on a pinch strength
Source: PLoS One. 2025 Jun 3;20(6):e0325359. doi: 10.1371/journal.pone.0325359 (PMC12133165; doi:10.1371/journal.pone.0325359)
Supplement: S3 Table — (DOCX) [file pone.0325359.s003.docx]

# Supporting Information

## S3 Table. Result of pinch strength ratio (flexion / extension) for each sex

|  | Pinch strength ratio (flexion / extension) | P-value |
| --- | --- | --- |
| Sex |  |  |
| Male | 1.40 ± 0.44 | 0.580 |
| Female | 1.32 ± 0.27 |  |
| Note: the pinch strength ratio between male and female was compared using a Student’s t-test. | | |
