# Supplementary material for: Ulnar finger posture effect on a pinch strength
Source: PLoS One. 2025 Jun 3;20(6):e0325359. doi: 10.1371/journal.pone.0325359 (PMC12133165; doi:10.1371/journal.pone.0325359)
Supplement: S4 Table — (DOCX) [file pone.0325359.s004.docx]

# Supporting Information

## S4 Table. Result of pinch strength for each trial

|  |  | Trials |  |  |
| --- | --- | --- | --- | --- |
| Ulnar finger posture | Hand dominance | Trial 1 | Trial 2 | Trial 3 |
| Flexion | Dominant | 4.8 ± 1.9 | 4.6 ± 1.8 | 4.6 ± 2.0 |
|  | Non-dominant | 4.4 ± 1.4 | 4.4 ± 1.6 | 4.3 ± 1.5 |
| Extension | Dominant | 3.7 ± 1.6 | 3.6 ± 1.4 | 3.5 ± 1.5 |
|  | Non-dominant | 3.3 ± 1.2 | 3.2 ± 1.0 | 3.0 ± 1.1 |
| Data are presented as mean ± standard deviation (SD). Unit of the pinch strength is kilograms of force (kgf). | | | | |
