# Supplementary material for: Ulnar finger posture effect on a pinch strength
Source: PLoS One. 2025 Jun 3;20(6):e0325359. doi: 10.1371/journal.pone.0325359 (PMC12133165; doi:10.1371/journal.pone.0325359)
Supplement: S5 Table — (DOCX) [file pone.0325359.s005.docx]

# Supporting Information

## S5 Table. Result of pinch strength for each examiner

|  |  | Examiner |  |
| --- | --- | --- | --- |
| Ulnar finger posture | Hand dominance | Examiner 1 | Examiner 2 |
| Flexion | Dominant | 4.6 ± 1.7 | 3.7 ± 1.6 |
|  | Non-dominant | 4.0 ± 1.4 | 3.1 ± 1.6 |
| Extension | Dominant | 3.5 ± 1.1 | 2.9 ± 0.8 |
|  | Non-dominant | 2.9 ± 0.8 | 2.5 ± 0.9 |
| Data are presented as mean ± standard deviation (SD). Unit of the pinch strength is kilograms of force (kgf). | | | |
