# Supplementary material for: Ulnar finger posture effect on a pinch strength
Source: PLoS One. 2025 Jun 3;20(6):e0325359. doi: 10.1371/journal.pone.0325359 (PMC12133165; doi:10.1371/journal.pone.0325359)
Supplement: S6 Table — (DOCX) [file pone.0325359.s006.docx]

# Supporting Information

## S6 Table. Result of pinch strength in test and retest

| Ulnar finger posture | Hand dominance | Test | Retest |
| --- | --- | --- | --- |
| Flexion | Dominant | 5.2 ± 1.8 | 4.9 ± 1.9 |
|  | Non-dominant | 4.6 ± 1.6 | 4.6 ± 2.0 |
| Extension | Dominant | 3.8 ± 0.9 | 3.6 ± 0.9 |
|  | Non-dominant | 3.2 ± 0.7 | 3.2 ± 0.9 |
| Data are presented as mean ± standard deviation (SD). Unit of the pinch strength is kilograms of force (kgf). | | | |
