# Supplementary material for: Ulnar finger posture effect on a pinch strength
Source: PLoS One. 2025 Jun 3;20(6):e0325359. doi: 10.1371/journal.pone.0325359 (PMC12133165; doi:10.1371/journal.pone.0325359)
Supplement: Attachment — Submitted filename: Response to reviewers.docx [file pone.0325359.s009.docx]

September 29. 2023

Dr. Saumya Srivastava

Academic Editor

*PLOS ONE*

Dear Dr. Srivastava;

On behalf of my coauthors, I thank you for the opportunity to revise and resubmit our manuscript **PONE-D-24-49287**, entitled **“Ulnar finger posture effect on a pinch strength.”** We found the Reviewers’ comments helpful in revising the manuscript and have carefully considered and responded to each suggestion. We have successfully incorporated the Reviewers’ feedback into our revised manuscript.

In our point-by-point response (presented below), the Reviewers’ comments are in Helvetica font and our responses are prefaced by “**RESPONSE**.” Corresponding changes are shown using the Track Changes option in the manuscript text in red text in the revised file, which was uploaded as a “Revised Article with Changes Highlighted” file. We have also uploaded a clean copy of our manuscript without the changes shown uploaded as our “Manuscript” file.

**Journal Requirements:**

1. Please ensure that your manuscript meets PLOS ONE's style requirements, including those for file naming.

**RESPONSE**: We prepared resubmitted manuscripts using the *PLOS ONE* style templates and ensured that all submissions met the *PLOS ONE* style requirements, including those for file naming.

2. We note that the grant information you provided in the ‘Funding Information’ and ‘Financial Disclosure’ sections do not match. When you resubmit, please ensure that you provide the correct grant numbers for the awards you received for your study in the ‘Funding Information’ section.

**RESPONSE**: We have provided incorrect grant numbers in the ‘Funding Information’ section. We have revised it accordingly (JP20K19344 and JP24K20530).

3. Thank you for stating the following financial disclosure: “The authors disclose the receipt of the following financial support for the research, authorship, and/or publication of this article: JSPS KAKENHI, Grant Number JP 20K19344 and 24K205300A supported this work.” Please state what role the funders took in the study. If the funders had no role, please state: "The funders had no role in study design, data collection and analysis, decision to publish, or preparation of the manuscript." If this statement is not correct you must amend it as needed. Please include this amended Role of Funder statement in your cover letter; we will change the online submission form on your behalf.

**RESPONSE**: We have revised the grant numbers (JP20K19344 and JP24K20530) and specified that the funders had no role in study design, data collection and analysis, decision to publish, or preparation in our cover letter as follows:

“This work was supported by Japan Society for Promotion of Science (JSPS) KAKENHI (Grant-in-Aid for Early-Career Scientists) awarded to S.D. [grant numbers JP20K19344 and JP24K20530]. JSPS website: [https://www.jsps.go.jp/english/]. The funders had no role in study design, data collection and analysis, decision to publish, or preparation of the manuscript.”

4. We note that your Data Availability Statement is currently as follows: “All relevant data are within the manuscript and in Supporting Information files.” Please confirm at this time whether or not your submission contains all raw data required to replicate the results of your study. Authors must share the “minimal data set” for their submission. PLOS defines the minimal data set to consist of the data required to replicate all study findings reported in the article, as well as related metadata and methods.

**RESPONSE**: We have added a dataset to Supplementary Information as ‘S1 Data. dataset.csv.’ This dataset contains all raw data required to replicate all study findings reported in the revised article.

5. When completing the data availability statement of the submission form, you indicated that you will make your data available on acceptance. We strongly recommend all authors decide on a data sharing plan before acceptance, as the process can be lengthy and hold up publication timelines. Please note that, though access restrictions are acceptable now, your entire data will need to be made freely accessible if your manuscript is accepted for publication. This policy applies to all data except where public deposition would breach compliance with the protocol approved by your research ethics board. If you are unable to adhere to our open data policy, please kindly revise your statement to explain your reasoning and we will seek the editor's input on an exemption. Please be assured that, once you have provided your new statement, the assessment of your exemption will not hold up the peer review process.

**RESPONSE**: As per the response to Comment 4 of the Journal Requirements, we have added a dataset to Supplementary Information as ‘S1 Data. dataset.csv.’ Our entire dataset is now freely accessible.

6. Please include your full ethics statement in the ‘Methods’ section of your manuscript file. In your statement, please include the full name of the IRB or ethics committee who approved or waived your study, as well as whether or not you obtained informed written or verbal consent. If consent was waived for your study, please include this information in your statement as well.

**RESPONSE**: We have included our full name of ethics committee and added the approval number in the ‘Materials and methods’ section of our manuscript file. Written informed consent was obtained from all participants before their participation. We have revised the manuscript as follows:

Lines 73–76: “Written informed consent was obtained from all participants before their participation. This study was approved by our institutional ethics review board (Ethics Committee for Epidemiology of Hiroshima University, approval number: E2018-1517) and conducted in accordance with the Declaration of Helsinki.”

7. Please review your reference list to ensure that it is complete and correct. If you have cited papers that have been retracted, please include the rationale for doing so in the manuscript text, or remove these references and replace them with relevant current references. Any changes to the reference list should be mentioned in the rebuttal letter that accompanies your revised manuscript. If you need to cite a retracted article, indicate the article’s retracted status in the References list and also include a citation and full reference for the retraction notice.

**RESPONSE**: We have ensured that our manuscript contains no retracted references. In addition, we have added following several references to the reference list in order to response to the Reviewers’ comments.

Additional references:

25.	Cohen J. Statistical Power Analysis for the Behavioral Sciences. 2nd ed. Lawrence Erlbaum Associates; 1988.33.

33.	Bland JM, Altman DG. Statistical methods for assessing agreement between two methods of clinical measurement. Lancet. 1986;1(8476):307-310. doi: 10.1016/S0140-6736(86)90837-835

34.	Iizuka T, Tomita Y. Reliability of motion phase identification for long-track speed skating using inertial measurement units. PeerJ. 2024;12:e18102. doi: 10.7717/peerj.18102.41

35.	Eto T, Kitamura S, Shikano A, Tanabe K, Higuchi S, Noi S. Estimating dim light melatonin onset time in children using delta changes in melatonin. Sleep Biol Rhythms. 2024;22(2):239-46. doi: 10.1007/s41105-023-00493-x.43

36.	Anvari A, Halpern EF, Samir AE. Essentials of Statistical Methods for Assessing Reliability and Agreement in Quantitative Imaging. Acad Radiol. 2018;25(3):391-396. doi: 10.1016/j.acra.2017.09.010.

41.	Aguiar LT, Martins JC, Lara EM, Albuquerque JA, Teixeira-Salmela LF, Faria CD. Dynamometry for the measurement of grip, pinch, and trunk muscles strength in subjects with subacute stroke: reliability and different number of trials. Braz J Phys Ther. 2016;20(5):395-404. doi: 10.1590/bjpt-rbf.2014.0173.

42.	Hock N, Lindstrom D. Normative data for the Baseline(R) 5 position hydraulic pinch meter and the relationship between lateral pinch strength and pinch span. J Hand Ther. 2021;34(3):453-62. doi: 10.1016/j.jht.2020.03.007.

43.	Dempsey PG, Ayoub MM. The influence of gender, grasp type, pinch width and wrist position on sustained pinch strength. International Journal of Industrial Ergonomics. 1996;17(3):259-73. doi: Doi 10.1016/0169-8141(94)00108-1.

44.	Shih YC, Ou YC. Influences of span and wrist posture on peak chuck pinch strength and time needed to reach peak strength. International Journal of Industrial Ergonomics. 2005;35(6):527-36. doi: 10.1016/j.ergon.2004.12.002.

45.	Heffernan C, Freivalds A. Optimum pinch grips in the handling of dies. Appl Ergon. 2000;31(4):409-14. doi: 10.1016/s0003-6870(99)00064-2.

**Additional Editor comments:**

The review process for your manuscript titled "Ulnar finger posture effect on a pinch strength" (Manuscript ID: [PONE-D-24-49287]) has been completed. The reviewers have suggested minor revisions to enhance the clarity and quality of your work.

Please find the reviewer's comments for your reference. Kindly revise the manuscript accordingly and submit the revised version along with a point-by-point response to the comments at your earliest convenience.

**RESPONSE**: We appreciate your revision of our manuscript and the opportunity to resubmit. We successfully incorporated the Reviewers’ feedback into our revised manuscript as discussed below.

**Review Comments to the Author**

Reviewer #1:

Thank you for the opportunity to review this interesting paper on finger postures during pinching. The experimental objectives, methods, and postoperative data evaluation methods including inter-rater evaluations are all well-documented. There are some questions regarding the discussion of the results. Please refer to the points below.

RESPONSE: We thank the Reviewer for these kind comments. We have revised our manuscript based on the Reviewer’s comments as described below.

COMMENT 1: Line 212, page16; The ICC values for inter-rater reliability (0.52-0.68) are not particularly high; please include a discussion addressing this point. Could this be due to a lack of experience on the part of Examiner 2?

RESPONSE: As the Reviewer pointed out, the lower ICC values for inter-rater reliability compared to intra-rater reliability (0.71–0.92) and test-retest reliability (0.71–0.91) could be due to difference in experience between Examiner 1 and Examiner 2, or to the shorter measurement training time (30 min) for Examiner 2. Our interpretation of these results is that it may be necessary for inexperienced examiners to be provided with sufficient measurement training (e.g., training time, measuring method instructions). We have added this in the ‘Discussion’ section as follows:

Lines 252–259: “On the other hand, the ICC values for inter-rater reliability (0.52–0.68) were lower than those for intra-rater (0.71–0.92) and test-retest (0.71–0.91) reliability. These lower ICC values for inter-rater reliability could be attributed to the difference in experience between Examiner 1 and Examiner 2, or to the insufficient measurement training time (30 min) for Examiner 2. It appears necessary for inexperienced examiners to receive more comprehensive measurement training, such as sessions longer than 30 min or instruction by a supervisor following the ASHT recommended measurement methods [1]”.

COMMENT 2: What are the causes of the differences in the natural ulnar finger posture during pinching? If pinch strength increases when the ring and little fingers are flexed posture, why do some individuals extend their ring and little fingers instead under power pulp pinch?

RESPONSE: People can develop various hand and finger posture during the growth stage, and ulnar finger extension in a pinch movement emerges as part of this process (Hohlstein, J *Occup Ther*, 1982). A previous study has suggested that the extension of the ulnar finger causes tension in the soft tissues around the index finger, leading to joint stability (Agee et al., *J Hand Surg Am*, 1980). However, the reason why some individuals extend the ulnar finger under power pulp pinch remains unclear. In future studies, we intend to examine this phenomenon from a biomechanical perspective to clarify why certain individuals extend the ulnar finger during pinching tasks. We have added the future prospects in the ‘Discussion’ section as follows:

Lines 285–289: “A detailed investigation using movement analysis and EMG would be required to clarify the exact influence of ulnar finger posture during pinching movements. These biomechanical and physiological measurements could also provide insight into why the ulnar finger is extended during pinching movements.”

Reviewer #2:

This manuscript is an important finding for hand therapy and outcome measurement assessment of the hand. I believe this work adds to the body of literature with new knowledge but it is not without its limitations. Please see my comments below.

RESPONSE: We thank the Reviewer for these kind comments. We have revised our manuscript based on the Reviewer’s comments, as described below.

COMMENT 1: Can you elaborate on why you chose to focus solely on pulp-to-pulp pinch strength rather than including other pinch types (e.g., lateral or tripod pinch) in this study? How might the inclusion of other pinch types alter the findings?

RESPONSE: As the Reviewer pointed out, this study focused on only pulp-to-pulp pinching and did not include measurements of the tripod pinching. This limitation was intentional to minimize participant fatigue and to accurately isolate the influence of ulnar finger posture during pinching tasks. As suggested by the Reviewer, future studies should measure the strength of tripod pinching measurements with appropriate experimental setup and additional conditions. Although the ulnar finger posture may affect lateral pinch strength, it is clinically uncommon for patients to perform the lateral pinch with the ulnar finger extended. Consequently, we did not compare the lateral pinching between flexion and extension of the ulnar fingers. We have revised the manuscript in the limitation paragraph of the ‘Discussion’ section as follows:

Lines 277–282: “Second, we only measured pulp-to-pulp pinching movements using the thumb and index finger. However, tripod pinching using the thumb, index, and middle fingers may also be influenced by the posture of the ring and little fingers. Future studies should compare the strength of the tripod pinch between flexion and extension of the ulnar fingers while ensuring experimental settings that minimize confounding factors such as examiner variability and fatigue.”

COMMENT 2: The abstract mentions no consensus on ulnar finger posture during pinch strength testing. Could you provide more context on the range of practices currently observed in clinical settings beyond the ASHT recommendation?

RESPONSE: We have added in the ‘Abstract’ that the ulnar finger differs in flexion and extension depending on the measurement method used in the previous study. In addition, we have also revised the ‘Introduction’ session of our manuscript as follows:

Abstract:

Lines 18–21: “…; however, there is no consensus regarding the posture of the three ulnar fingers during pinch strength testing, and existing studies have different flexion or extension posture, depending on the measurement method used”.

Introduction:

Lines 52–54: “…, and depending on the measurement method used, the posture of the ulnar fingers differs in flexion or extension [7, 15-19].”

COMMENT 3: How do you envision these findings being integrated into standardized clinical protocols for pinch strength assessment, given the variability in current practices?

RESPONSE: Since our findings enhance the ASSH’s recommended measurement methods, clinicians should follow a standardized clinical protocol that is integrated into this recommendation. Furthermore, based on the results of this study, which showed moderate ICC for the inter-rater reliability, the same examiner should perform the pinch testing when longitudinally assessing a patient’s pinch strength. We have created a new paragraph in the ‘Discussion’ section to highlight the clinical relevance as follows:

Lines 260–267: “Our findings support the results of a previous study on the effect of ulnar finger posture during the pinching movement [12] and could be integrated into standard protocol of the ASSH's recommended measurement methods [1]. Based on our findings, clinicians should consistently instruct patients to flex their ulnar fingers during pinch testing as the flexion posture generates greater pinch strength and exhibits less inter-, intra-rater, and test-retest variability than the extension posture. Furthermore, given the moderate ICC for the inter-rater reliability, having the same examiner perform pinch testing when longitudinally assessing a patient’s pinch strength is advisable.”

COMMENT 4: You note that the effect of ulnar finger posture on pinch strength is not well explored. Are there specific biomechanical or physiological findings/issues from prior studies that guided your research question?

RESPONSE: We are guided in our research questions on the effect of ulnar finger posture by biomechanical findings. We have added our consideration of the biomechanical findings in the ‘Introduction’ section as follows:

Lines 54–57: “Considering the biomechanical findings that the agonist (e.g., digitorum superficialis) and antagonist muscles (e.g., extensor digitorum) involved in index finger flexion share a muscle belly with the four fingers, ulnar finger posture would affect the strength of the maximum pulp pinching between the thumb and index finger [20-23]”.

COMMENT 5: Participants were instructed to extend their ulnar fingers "naturally" in the extension posture. How was "natural" extension defined or ensured during testing, and did this vary significantly between participants?

RESPONSE: We did not define the degree of ulnar finger extension, but instead instructed participants to extend their ulnar fingers naturally in the extension posture, since we wanted to examine the effect of ulnar finger extension during the pinching movement in a more clinical and practical situation. Additionally, we did not measure angles of the ulnar finger joints, so it was unclear if the degree of “natural” extension varied significantly between participants. We have revised the manuscript in the ‘Materials and methods’ section to address why we did not objectively define the degree of ulnar finger extension as follows:

Lines 86–91: “Two ulnar finger postures were used, namely flexion and extension. The metacarpophalangeal and interphalangeal joints of the ulnar fingers were fully flexed in the flexion posture and were naturally extended in the extension posture. Regarding the extension posture, the participants were not instructed on the degree of extension of the joints to simulate a more clinical and practical pinch testing situation (Fig 1B).”

COMMENT 6: The sample size was determined using a power analysis with an effect size of 0.25. Can you explain how this effect size was chosen, and whether it aligns with effect sizes reported in prior pinch strength studies?

RESPONSE: The effect size was not referenced in reports in prior pinch strength studies and was instead based on the medium effect size (0.25) reported by Cohen (Cohen, *book “Statistical Power Analysis for the Behavioral Sciences”*, 1988). Although our sample size (n = 33) was comparable to that of several previous pinch strength studies (MacDermid et al., *J Hand Ther*, 1994, n = 35; Mathiowetz et al., *J Hand Surg Am*, 1984, n = 27; Pryce, *J Biomech*, 1980, n = 30; Schreuders et al., *Physical Therapy*, 2003, n = 33), it was considered that the small sample size restricted the generalizability of our results as described in the limitation paragraph of our manuscript (Line 273–277). We have revised the manuscript as follows:

Lines 68–70: “… which was determined to be 30 participants (medium effect size = 0.25; α = 0.05; power = 0.90) [25]. In total, 33 healthy volunteers (19 males and 14 females) with a mean age of 34.0 ± 13.6 years were recruited.”

COMMENT 7: Examiner 2 had less experience and received 30 minutes of training. Did you assess whether this training was sufficient to minimize systematic differences between examiners, beyond what the inter-rater reliability results show?

RESPONSE: We examined the presence or absence of systematic errors (additive and proportional errors) to determine the degree of inter-rater variability. As a result, we found no significant systematic errors among them. Therefore, the degree of variability may be within acceptable limits. However, the difference in experience between Experimenter 1 and Experimenter 2 and the short training time may have affected. We have revised Table 3 and also added additional data analysis, results, and consideration to the ‘Materials and methods’, ‘Results’, and ‘Discussion’ sections, respectively as follows:

Materials and methods:

Lines 152–160: “Additionally, to examine the presence or absence of systematic errors in the differences between the intra- and inter-rater and test-retest measurements, additive and proportional errors were evaluated [33-36]. Additive error was evaluated by calculating the limit of agreement (LoA) using the 95% CI of the mean of the difference between the two measurements [34]. We considered the additive error to be absent when the LoA had intervals that included zero. Proportional error was evaluated by calculating a regression coefficient (r) of the relationship between the two measurements [36]. We considered the proportional error to be absent when the correlation was not statistically significant [33-35].”

Results:

Lines 184–185: “An overview of the ICC, 95% CI, SEM, MDC95%, LoA, and correlation coefficient (r) for intra- and inter-rater and test-retest reliability is listed in Table 3.”

Lines 194–195: “All the LoA had intervals that included zero, and none of the correlations coefficients (r values) were statistically significant.”

Discussion:

Lines 238–241: “Additionally, all the LoA had intervals that included zero, indicating the absence of additive error, and the correlations coefficients (r values) were not significant, suggesting the absence of proportional error. These results mean that the systematic error was within acceptable limits.”

Lines 252–259: “On the other hand, the ICC values for inter-rater reliability (0.52–0.68) were lower than those for intra-rater (0.71–0.92) and test-retest (0.71–0.91) reliability. These lower ICC values for inter-rater reliability could be attributed to the difference in experience between Examiner 1 and Examiner 2, or to the insufficient measurement training time (30 min) for Examiner 2. It appears necessary for inexperienced examiners to receive more comprehensive measurement training, such as sessions longer than 30 min or instruction by a supervisor following the ASHT recommended measurement methods [1].”

COMMENT 8: Pinch strength was measured with a 7mm pinch width. Why this specific width? How might varying pinch widths affect the observed differences between flexion and extension postures?

RESPONSE: The pinch width of the pinch gauge used in this study was fixed at 7 mm, and the width could not be adjusted for each individual. Although the pinch width that results in the greatest pinch strength varies across previous studies (Hock et al., *J Hand Ther*, 2021; Dempsey et al., *Int J Ind Ergon*, 1996; Imrhan et al., *Int J Ind Ergon*, 1995; Shih et al., *Int J Ind Ergon*, 2004; Heffernan et al., *Appl Ergon*, 2000) and remains inconclusive, pinch width could influence pinch strength. From biomechanical considerations, it is suspected that in the extension posture of this study, a larger pinch width could result in greater pinch strength because the ulnar finger extension involuntary exerts force in the direction of extension of the index finger and impeding the index finger flexion. We have addressed the effect of pinch width on pinch strength to the limitations paragraph in ‘Discussion’ section regarding the biomechanical changes in the balance between flexor and extensor muscles as follows:

Lines 289–294: “Finally, the pinch width was fixed at 7 mm due to equipment constraints, and the width could not be adjusted for each individual. Different pinch widths can increase or decrease pinch strength [8, 42-45]. In the extension posture used in this study, a larger pinch width may result in greater pinch strength because involuntary extension force applied to the index finger is weakened with increasing pinch width.”

COMMENT 9: Pinch strength in the flexion posture was 1.34 times greater than in the extension posture. Did you explore whether this ratio varied significantly across individuals or subgroups (e.g., by age, hand size, or sex)?

RESPONSE: We conducted a subgroup analysis to examine whether participants’ demographic data, including age, hand size, and sex, influence the pinch strength ratio. Our analysis indicated that the pinch strength ratio did not vary with these variables. This analysis and its results have been added to the ‘Materials and methods (statistical analysis)’ and the ‘Results’ sections, and Supporting Information (S2 and S3).

Lines 136–141: “As a subgroup analysis to investigate whether pinch strength in the ulnar finger position changes based on participant’s demographic data, the relationship between pinch strength ratio between the flexion and extension postures (flexion relative to extension) and age, height, body mass, and hand size was examined using a Pearson's correlation coefficient. Furthermore, the pinch strength ratio between male and female was compared using a Student’s t-test.”

Lines 176–178: “Subgroup analysis indicated that the pinch strength ratio between the flexion and extension postures did not statistically vary by age (p = 0.779), height (p = 0.398), body mass (p = 0.68), hand sizes (p = 0.186–0.945), or sex (p = 0.580) (S2, S3).”

COMMENT 10: Also, the natural ulnar finger posture was flexion in 66.7% of participants. Did you analyze whether participants with a natural extension posture exhibited different pinch strength patterns compared to those with a natural flexion posture?

RESPONSE: This aspect was analyzed, yes. The participants with a natural extension posture did not show different pinch strength patterns when compared to participants with a natural flexion posture. As noted in S1 Table, the pinch strength in the flexion posture was greater than that in the extension posture for participants in both the natural extension and flexion posture groups. We have revised the manuscript to enhance clarity as follows:

Lines 165–167: “Pinch strength between participants with the natural flexion and extension ulnar finger postures did not differ significantly (S1 Table).”

COMMENT 11: Inter-rater reliability was moderate (ICC: 0.52–0.68), lower than intra-rater and test-retest reliability. What factors do you think contributed to this, and how might they be addressed in future studies?

RESPONSE: One of the factors that the ICC values for inter-rater reliability were lower compared to intra-rater reliability (0.71–0.92) and test-retest reliability (0.71–0.91) could be due to difference in experience between Examiner 1 and Examiner 2. Our interpretation of these results is that it should be necessary for inexperienced examiners to receive more comprehensive measurement training, such as sessions longer than 30 min or instruction by a supervisor following the ASHT recommended measurement methods. We have added this in the paragraph on clinical relevance in the “Discussion” section.

Furthermore, an additional statistical analysis was performed to assess the degree of variability in intra-rater, inter-rater, and test-retest reliability. As a result, we found that there were no significant systematic errors (additive and proportional errors) among them. Therefore, the degree of variability, including the inter-rater variability, may be acceptable. We have added the additional data analysis, results, and consideration to the ‘Materials and methods’, ‘Results’, and ‘Discussion’ sections, respectively. We have also revised Table 3.

Materials and methods:

Lines 152–160: “Additionally, to examine the presence or absence of systematic errors in the differences between the intra- and inter-rater and test-retest measurements, additive and proportional errors were evaluated [33-36]. Additive error was evaluated by calculating the limit of agreement (LoA) using the 95% CI of the mean of the difference between the two measurements [34]. We considered the additive error to be absent when the LoA had intervals that included zero. Proportional error was evaluated by calculating a regression coefficient (r) of the relationship between the two measurements [36]. We considered the proportional error to be absent when the correlation was not statistically significant [33-35].”

Results:

Lines 184–185: “An overview of the ICC, 95% CI, SEM, MDC95%, LoA, and correlation coefficient (r) for intra- and inter-rater and test-retest reliability is listed in Table 3.”

Lines 194–195: “All the LoA had intervals that included zero, and none of the correlation coefficients (r values) were statistically significant.”

Discussion:

Lines 238–241: “Additionally, all the LoA had intervals that included zero, indicating the absence of additive error, and the correlations coefficients (r values) were not significant, suggesting the absence of proportional error. These results mean that the systematic error was within acceptable limits.”

Lines 252–259: On the other hand, the ICC values for inter-rater reliability (0.52–0.68) were lower than those for intra-rater (0.71–0.92) and test-retest (0.71–0.91) reliability. These lower ICC values for inter-rater reliability could be attributed to the difference in experience between Examiner 1 and Examiner 2, or to the insufficient measurement training time (30 min) for Examiner 2. It appears necessary for inexperienced examiners to receive more comprehensive measurement training, such as sessions longer than 30 min or instruction by a supervisor following the ASHT recommended measurement methods [1].”

COMMENT 12: The SEM and MDC95% values were provided for reliability. How do these values translate to clinically meaningful differences in pinch strength, for patient populations with hand impairments for example.

RESPONSE: Since clinicians need to assess whether patients with hand impairments improve after intervention, we should focus on the SEM and MCD_95%_ values for the test-retest the reliability. Particularly, it would be clinically and practically important to focus on the MDC_95%_ in the test-retest reliability because the MDC_95%_ represents real change beyond measurement error. In this study, the MDC_95%_ values for the flexion posture in test-retest reliability were 2.12 kg for the dominant hand and 1.51 kg for the non-dominant hand. This is consistent with the MCD_95%_ results for pulp pinch in a previous study investigating test-retest reliability in paretic and non-paretic upper limbs of stroke patients (paralyzed upper extremity: 1.36–1.73 kg; non-paretic upper extremity: 1.38–2.20 kg) (Aguiar et al., *Braz J Phys Ther*, 2016.). Thus, when assessing changes in a longitudinal study, a change of approximately 2 kg or more could be interpreted as a meaningful improvement. We have added a new paragraph in the ‘Discussion’ section on how the MDC_95%_ value translates into a clinically meaningful difference. as follows:

Lines 267–272: “In addition, when assessing longitudinally, a change in pinch strength of more than 2 kg can be interpreted as an improvement, consistent with a previous study [41], because MDC_95%_ represents true change beyond measurement error and the MDC_95%_ results for test-retest reliability of flexion posture in this study was around 2 kg (2.12 kg for the dominant hand, 1.51 kg for the non-dominant hand).”

In addition to the Editor’s and Reviewers’ Comments, minor corrections were made to the following text [*with reason for correction*]:

1. In Line 24–25: “... was tested in two positions (flexion and extension)” [*To improve consistency of the manuscript*]
2. In Line 196, Table 3: “...; ICC = intraclass correlation coefficient; ...” [*Misspelling*]
